# Around the Hospitals

**Published:** 1893-07-08

**Authors:** 


### Around the Hospitals.

Notice to the Managers of Institutions.—Special space is now reserved for the insertion of notices of hospital meetings and festivals. The Editor requests that all notices may reach The Hospital Office, 428, Strand, at least one week before the date of each meeting.

**The Gravesend Hospital.**—The hospital has so amply justified the necessity for its existence that it is hoped it will be found possible before long to provide extended accommodation for in-patients. Several large sums of money have already been promised towards this end. During the past year 313 patients were admitted, and 4,386 out patients were treated, coming from no less than 51 different districts, representing a very wide area for the ministrations of a small hospital. The number of out-patients treated during the past year largely exceeds that of any previous year.

